# Concerns the publication: Vitale SG, Giannini A, Carugno J et al. Hysteroscopy: where did we start, and where are we now? The compelling story of what many considered the “Cinderella” of gynecological endoscopy. Arch Gynecol Obstet 310, 1877–1888 (2024)

**DOI:** 10.1007/s00404-024-07868-6

**Published:** 2024-12-09

**Authors:** M. David

**Affiliations:** Charité - Universitätsmedizin Berlin, Berlin, Germany

Dear Sir, 

It is always gratifying to read an article in a gynecological-obstetrical journal that deals with the historical development of diagnostic measures, instruments, procedures, operations. In this respect, the work of Vitale et al. in the Archive 2024 on a “stepchild” of gynecological operations, hysteroscopy, is very welcome. This article also makes it clear that this valuable surgical technique has many fathers. It is interesting that a review by a British-Greek group of authors on the “History of hysteroscopy as an endoscopic method” [3] was published in another journal at the same time. Here, as in an older review by [4] on the history of hysteroscopy in the nineteenth and twentieth centuries, at least Mikulicz-Radecki is mentioned with his important publication from 1927, but also in the multiple reviews by [1] cited the work of [2] from “Fertility Sterility”. However, [1] does not mention the works of [5–7].

This may be due to the fact that the literature compilation by Vitale et al. From a methodological point of view, this is not a systematic review, but rather a narrative review. As is standard practice, authors have discretion and flexibility in selecting the literature sources used in the article. The medical-historical description of developments in hysteroscopy was obviously not intended to be complete. It is all the more necessary to point out the works published in the “Archive for Gynecology” in the first third of the twentieth century.

This is all the more regrettable since two of these works (Gauß; Schroeder) appeared in this journal, the “Archiv”. Mikulicz-Radecki and his colleague A. Freund also published a short article on (tubal) hysteroscopy in the “Archiv”. Perhaps this “overlook” is related to the fact that the “Archiv” was still a German-language journal at the time. It would be unfortunate if this were the reason not to mention these important works.
